# MSSA Outbreak detected with software combining microbial resistance pattern and location within hospital

**DOI:** 10.1186/1753-6561-5-S6-P31

**Published:** 2011-06-29

**Authors:** P Gruteke, E Walgreen, CJ van der Mark

**Affiliations:** 1 OLVG, Amsterdam, Netherlands; 2 Flevoziekenhuis, Almere, Netherlands

## Introduction / objectives

Clinical microbiology laboratories are increasing in size, providing services to several hospitals. Clusters of infections caused by other than readily recognized organisms such as MRSA, might easily go undetected. Dedicated software should overcome this problem.

## Methods

A new module in the laboratory information system GLIMS by MIPS Belgium, combines isolate resistance data with patient location data derived from the hospital information system. Antimicrobial resistance patterns of new clinical isolates are compared with previous patterns, and identical resistance patterns are checked at ward level for simultaneous patient stay. If these conditions are met, a report with isolate, details of the two patients and both time and location of possible transfer is generated. Before sending the report to the infection control practitioner, common resistance patterns are filterd to improve the noise to signal ratio.

## Results

The microbiology laboratory working for 3 hospitals introduced the system in June 2010. From late January 2011 to early March reports from a surgical ward in the Flevoziekenhuis noted MSSA isolates resistant to ciprofloxacin, fusidic acid, erythromycin and clindamycin. The number of involved patients mounted to 12. Nursing practices were audited, and nurses with skin lesions were cultured. No obvious breach in infection control measures or healtcare worker sources were detected. Reinforcement of exsisting hygiene procedures was sufficient to stop the transmission.

## Conclusion

As clinical microbiology services tend to be concentrated for efficiency reasons, manual surveillance of resistance patterns becomes less feasible. Our GLIMS module proved useful in detecting a cluster of MSSA transmission in a surgical ward.

## Disclosure of interest

None declared.

